# Unveiling the Clinical Path of Microinvasive Breast Cancer: A Comparative Study With Tis‐T1 Breast Cancer

**DOI:** 10.1002/cam4.70297

**Published:** 2024-10-09

**Authors:** Ran Song, Dong‐Eun Lee, So‐Youn Jung, Seeyoun Lee, Han‐Sung Kang, Jai Hong Han, Jaeyeon Woo, Eun‐Gyeong Lee

**Affiliations:** ^1^ Department of Surgery Konyang University Hospital, Konyang University College of Medicine Daejeon Republic of Korea; ^2^ Biostatistics Collaboration Team, Research Core Center Research Institute of National Cancer Center Goyang Republic of Korea; ^3^ Department of Surgery, Center of Breast Cancer National Cancer Center Goyang Republic of Korea

**Keywords:** breast cancer, carcinoma in situ, invasive carcinoma, microinvasion, prognosis, recurrence

## Abstract

**Purpose:**

The prognosis of microinvasive breast cancer (MIBC) is controversial, with a high reported rate of local recurrence (LR). This study aimed to evaluate the characteristics, treatments, and prognosis of patients with MIBC compared to those with carcinoma in situ (CIS) or early invasive cancer.

**Methods:**

Patients who diagnosed with CIS or stage I breast cancer were retrospectively enrolled. Using the Kaplan–Meier method, local recurrence‐free survival (LRFS), systemic recurrence‐free survival (SRFS), and cancer‐specific survival (CSS) were compared according to T stage. The prognostic factors associated with LRFS were identified using the Cox proportional hazards model.

**Results:**

According to T stage, 517 (21.6%), 200 (8.4%), 207 (8.7%), 363 (15.2%), and 1101 (46.1%) patients had Tis, T1mi, T1a, T1b, and T1c tumors, respectively. The proportion of human epidermal growth factor receptor 2‐positive tumors was significantly higher in patients with MIBC (*p* < 0.0001). The administered adjuvant treatments also showed differences according to T stage (*p* < 0.0001). During the 73‐month median follow‐up period, patients with MIBC showed significantly worse LRFS than those with T1a or T1c tumors (*p* = 0.002). There was no significant difference in SRFS and CSS. In the Cox regression analysis, tumor multiplicity (*p* = 0.017), Ki‐67 (*p* = 0.025), cancer subtype (*p* = 0.034), adjuvant endocrine therapy (*p* = 0.003), and adjuvant radiation therapy (*p* < 0.0001) were significant prognostic factors associated with LRFS.

**Conclusion:**

The risk of LR was higher in patients with MIBC than in those with small invasive breast cancer. Therefore, if indicated, adjuvant endocrine and radiation therapies should be administered to prevent undertreatment in patients with MIBC.

## 1 Introduction

With the increasing rate of breast cancer screening and development in screening equipment, the diagnosis of early stage breast cancer continues to improve [1, 2]. According to recent statistics, the incidence of local‐stage breast cancer in the United States increases by 0.9% per year [3]. In Korea, stage 0 or I breast cancer accounts for approximately 61.6% of entire breast cancer [4].

Microinvasive breast cancer (MIBC) is defined by the current American Joint Committee on Cancer staging manual as the extension of breast cancer cells beyond the basement membrane with a maximum invasive focus of no more than 0.1 cm in the great dimension [5]. MIBC is a rare disease entity, comprising only 0.68%–2.4% of all breast cancers [6]. Therefore, research on the treatment and clinical prognosis of MIBC remains limited.

Previous studies have reported contradictory results regarding the prognosis of MIBC. Some studies have reported that MIBC has a similar prognosis to carcinoma in situ (CIS) [7, 8, 9], while others have reported that MIBC has a worse prognosis compared to CIS and similar survival rates to invasive cancer [10, 11]. In recent studies, MIBC has a higher local recurrence (LR) rate than invasive cancer or CIS without microinvasion, despite no difference in overall survival [12, 13].

According to current guidelines, systemic chemotherapy or targeted therapy is generally not recommended for MIBC because of the good survival rate and minimal benefits of treatment [14]. However, an accurate understanding of the characteristics and prognosis of MIBC is necessary to prevent undertreatment and provide appropriate treatment for these patients.

This study aimed to evaluate the pathological characteristics, treatments, and prognostic outcomes of patients with MIBC compared to those with CIS or early invasive breast cancer. In addition, we aimed to identify prognostic factors associated with survival.

## 2 Materials and Methods

### 2.1 Patient Selection

Patients histopathologically diagnosed with CIS or stage I (pTis‐T1cN0) breast cancer after curative surgery at the National Cancer Center, Korea, between January 2012 and December 2017 were retrospectively enrolled in this study. Patients who met the following criteria were excluded: (1) male sex; (2) previous history of breast cancer; (3) diagnosis of bilateral breast cancer; (4) administration of neoadjuvant chemotherapy; (5) diagnosis of lobular carcinoma in situ and (6) pathological confirmation of lymph node (LN)‐positive status in surgically excised specimens, including sentinel LNs.

### 2.2 Pathological Examination

The pathological reports of all patients were reviewed. MIBC was diagnosed when the size of cancer cell invasion beyond the basement membrane ≤ 1 mm in the longest diameter. Tumor size was measured as the size of the largest invasive focus in T1a–c tumors, and as the size of the largest lesion, either invasive focus or in situ component, in Tis and T1mi tumors. The histologic features included the type and grade. Ki‐67 was presented as a percentage between 0% and 100%. Cancer subtypes were classified according to the expression of estrogen receptor (ER), progesterone receptor (PR), and human epidermal growth factor receptor 2 (HER2). In immunohistochemical staining, ER and PR were evaluated using an Allred score (0–8), and a score ≥ 3 was considered as positive. HER2 status was reported as positive when scores were 3+ or 2+ with a positive result of in situ hybridization.

### 2.3 Patient Follow‐Up and Outcomes

Patients were administered adjuvant treatment, such as chemotherapy, endocrine therapy, or radiation therapy, based on the clinician's decision after surgery. They were assessed every 6 months for 5 years. Thereafter, the follow‐up intervals varied according to the patients' clinical status. Image screening was performed every 6 months or 1 year, and bilateral mammograms or breast magnetic resonance imaging were obtained for breast and axillary evaluation. Chest computed tomography (CT), abdominopelvic CT, and whole‐body bone scans were obtained for evaluation of systemic recurrence.

LR was defined as the histopathological confirmation of invasive or in situ cancer in the ipsilateral breast or regional LNs. Systemic recurrence was defined as the confirmation of cancer by imaging or histopathology in other organs beyond the ipsilateral breast or regional LNs, including the bone, lungs, liver, and brain.

### 2.4 Statistical Analysis

Patients with MIBC were compared to those with CIS or T1a–c tumors. Chi‐squared tests were used to compare the characteristics of each T stage. Local recurrence‐free survival (LRFS) was defined as the time from the date of surgery to LR. Systemic recurrence‐free survival (SRFS) was defined as the time from the date of surgery to systemic recurrence. Cancer‐specific survival (CSS) was defined as the time from the date of surgery to death from breast cancer. Patients were censored at the last follow‐up. Survival curves were estimated using the Kaplan–Meier method and compared using the log‐rank test. A Cox proportional hazards model was used to identify factors associated with survival. Multivariable Cox proportional hazard analysis was performed using variables with *p* < 0.1 in the univariable analysis, employing the backward selection method with an elimination criterion of *p* < 0.05. *p*‐values (*p*) less than 0.050 were considered statistically significant. All statistical analyses were performed using R software (version 4.2.1; R Foundation for Statistical Computing, Vienna, Austria).

## 3 Results

In total, 2740 patients diagnosed with pTis‐T1cN0 breast cancer were enrolled in this study. Of these, 2388 were finally included in the analysis after excluding those who met the exclusion criteria. The baseline characteristics of the patients in each T stage are presented in Table 1. The patient distribution across the different tumor stages was as follows: Tis (*n* = 517), T1mi (*n* = 200), T1a (*n* = 207), T1b (*n* = 363), and T1c (*n* = 1101). The mean patient age was 51.0 ± 10.4 years, and 53.7% were premenopausal. The median tumor size of the lesion, either invasive focus or in situ component, was 1.2 cm (range, 0.1–11.0 cm), and 28.6% were of histologic grade 3. Among the 517 patients with CIS, 468 (90.5%) were ductal carcinoma in situ (DCIS), 45 (8.7%) were papillary carcinoma in situ, 3 (0.6%) were intracystic papillary carcinoma, and 1 (0.2%) was Paget's disease. All patients with MIBC had ductal type cancer. Among the patients with T1a–c tumors, 87.7% (1465/1671) were invasive ductal carcinoma, 4.2% (70/1671) were invasive lobular carcinoma, 2.8% (46/1671) were mucinous carcinoma, and 1.9% (32/1671) were tubular carcinoma. Other histological features included cribriform carcinoma (26/1671), papillary carcinoma (14/1671), metaplastic carcinoma (13/1671), apocrine carcinoma (3/1671), secretory carcinoma (1/1671), and lymphoepithelioma‐like carcinoma (1/1671).

**TABLE 1:** Clinicopathological characteristics of study population.

Characteristics	Total (*n* = 2388)	Tis (*n* = 517)	T1mi (*n* = 200)	T1a (*n* = 207)	T1b (*n* = 363)	T1c (*n* = 1101)
Age (year)						
Mean ± SD	51.0 ± 10.4	50.1 ± 10.3	51.0 ± 10.7	49.6 ± 9.7	50.6 ± 9.7	51.9 ± 10.7
≤ 40	338 (14.2%)	79 (15.3%)	36 (18%)	34 (16.4%)	55 (15.2%)	134 (12.2%)
> 40	2050 (85.9%)	438 (84.7%)	164 (82%)	173 (83.6%)	308 (84.9%)	967 (87.8%)
Menopausal status						
Premenopausal	1282 (53.7%)	298 (57.6%)	99 (49.5%)	117 (56.5%)	207 (57%)	561 (51%)
Postmenopausal	1106 (46.3%)	219 (42.4%)	101 (50.5%)	90 (43.5%)	156 (43%)	540 (49.1%)
Type of surgery						
BCS	2059 (86.2%)	417 (80.7%)	146 (73%)	166 (80.2%)	321 (88.4%)	1009 (91.6%)
Mastectomy	329 (13.8%)	100 (19.3%)	54 (27%)	41 (19.8%)	42 (11.6%)	92 (8.4%)
Tumor multiplicity						
Single	2121 (88.8%)	478 (92.5%)	180 (90%)	177 (85.5%)	315 (86.8%)	971 (88.2%)
Multiple (≥ 2)	267 (11.2%)	39 (7.5%)	20 (10%)	30 (14.5%)	48 (13.2%)	130 (11.8%)
Tumor size^a^ (cm)						
Mean ± SD	1.4 ± 1.1	2.2 ± 1.8	3.4 ± 2.1	0.4 ± 0.1	0.8 ± 0.1	1.5 ± 0.3
Median (min–max)	1.2 (0.1–11.0)	1.7 (0.1–11)	3.0 (0.1–9.0)	0.4 (0.1–0.8)	0.8 (0.5–1.7)	1.5 (1.1–2.0)
Histologic type						
DCIS	468 (19.6%)	468 (90.5%)	N/A	N/A	N/A	N/A
Other CIS	49 (2.1%)	49 (9.5%)	N/A	N/A	N/A	N/A
IDC	1665 (69.7%)	N/A	200 (100%)	187 (90.3%)	312 (86%)	966 (87.7%)
ILC	70 (2.9%)	N/A	0 (0%)	10 (4.8%)	15 (4.1%)	45 (4.1%)
Other invasive carcinoma	136 (5.7%)	N/A	0 (0%)	10 (4.8%)	36 (9.9%)	90 (8.2%)
Histologic grade (*n* = 1856)						
1	317 (17.1%)	N/A	14 (7%)	52 (25.1%)	101 (27.8%)	150 (13.6%)
2	1008 (54.3%)	N/A	91 (45.5%)	108 (52.2%)	198 (54.6%)	611 (55.5%)
3	531 (28.6%)	N/A	82 (41%)	47 (22.7%)	63 (17.4%)	339 (30.8%)
Ki‐67 (%) (*n* = 2370)						
Median (min–max)	16.0 (< 1.0–96.0)	9.0 (< 1.0–77.0)	23.0 (1.0–67.0)	13.0 (1.0–92.0)	15.0 (1.0–96.0)	20.0 (1.0–95.0)
≤ 14	1106 (46.7%)	335 (66.3%)	60 (30%)	108 (52.9%)	177 (49%)	426 (38.7%)
> 14	1264 (53.3%)	170 (33.7%)	140 (70%)	96 (47.1%)	184 (51%)	674 (61.3%)

Abbreviations: BCS, breast‐conserving surgery; CIS, carcinoma in situ; DCIS, ductal carcinoma in situ; IDC, invasive ductal carcinoma; ILC, invasive lobular carcinoma; max, maximum; min, minimum; N/A, non‐applicable; SD, standard deviation.

^a^ Tumor size was measured as the size of the invasive focus in T1a–c tumors, and as the size of the largest lesion, either invasive focus or in situ component, in Tis and T1mi tumors.

Table 2 presents the cancer subtypes and adjuvant treatment administered according to T stage. Among the total study population, 1865 (78.2%) patients were ER‐positive, 1637 (68.6%) were PR‐positive, and 451 (18.9%) HER2‐positive. The receptor status of patients with MIBC was as follows: 109 (54.5%) were ER‐positive, 93 (46.5%) were PR‐positive, and 104 (52%) were HER2‐positive. When comparing the cancer subtypes of MIBC with other T stages, ER/PR+ and HER2− tumors showed a lower proportion of 40.0% in MIBC, whereas HER2+ tumors, including ER/PR+ and HER2+, or ER/PR− and HER2+ subtypes, showed higher proportions of 17.0% and 35.0%, respectively. These differences in the subtypes were statistically significant (*p* < 0.0001). Regarding adjuvant treatment, 25.1% of the patients received chemotherapy and 7.4% received anti‐HER2 targeted therapy; all patients were diagnosed with invasive cancer. Endocrine and radiation therapies were administered to 75.5% and 78.6% of total patients, respectively. Radiotherapy was administered to 90.2% (1857/2059) of patients who underwent breast‐conserving surgery (BCS). According to T stage, the rates in these patients were 70.0% (292/417), 97.3% (142/146), 94.6% (157/166), 95.6% (307/321), and 95.0% (959/1009) for Tis, T1mi, T1a, T1b, and T1c tumors, respectively. Among the 329 patients who underwent mastectomy, 21 (6.4%) received radiotherapy. The rates of radiotherapy were 1.0% (1/100), 9.3% (5/54), 9.8% (4/41), 9.5% (4/42), and 7.6% (7/92) for Tis, T1mi, T1a, T1b, and T1c tumors, respectively. All patients who underwent adjuvant chemotherapy or anti‐HER2 therapy were at stage T1a or above, and the proportion of patients receiving treatment increased with the advancement of T stage. The administered adjuvant treatments significantly differed according to T stage (*p* < 0.0001).

**TABLE 2:** Breast cancer subtypes and adjuvant treatment according to T stage.

Characteristics	Total (*n* = 2388)	Tis (*n* = 517)	T1mi (*n* = 200)	T1a (*n* = 207)	T1b (*n* = 363)	T1c (*n* = 1101)	*p*
Subtype (*n* = 2384)							< 0.0001
ER/PR+ HER2−	1691 (70.9%)	383 (74.7%)	80 (40.0%)	161 (77.8%)	283 (78.0%)	784 (71.2%)	
ER/PR+ HER2+	203 (8.5%)	46 (9.0%)	34 (17.0%)	11 (5.3%)	22 (6.1%)	90 (8.2%)	
ER/PR− HER2+	248 (10.4%)	57 (11.1%)	70 (35.0%)	25 (12.1%)	19 (5.2%)	77 (7.0%)	
ER/PR− HER2−	242 (10.2%)	27 (5.3%)	16 (8.0%)	10 (4.8%)	39 (10.7%)	150 (13.6%)	
Chemotherapy							< 0.0001
No	1788 (74.9%)	517 (100%)	200 (100%)	204 (98.6%)	299 (82.4%)	569 (51.7%)	
Yes	600 (25.1%)	0	0	3 (1.5%)	64 (17.6%)	532 (48.3%)	
Endocrine therapy							< 0.0001
No	584 (24.5%)	164 (31.7%)	88 (44.0%)	35 (16.9%)	58 (16.0%)	239 (21.7%)	
Yes	1804 (75.5%)	353 (68.3%)	112 (56.0%)	172 (83.1%)	305 (84.0%)	862 (78.3%)	
Anti‐HER2 therapy							< 0.0001
No	2212 (92.6%)	517 (100%)	200 (100%)	206 (99.5%)	348 (95.9%)	941 (85.5%)	
Yes	176 (7.4%)	0	0	1 (0.5%)	15 (4.1%)	160 (14.5%)	
Radiation therapy							< 0.0001
No	510 (21.4%)	224 (43.3%)	53 (26.5%)	46 (22.2%)	52 (14.3%)	135 (12.3%)	
Yes	1878 (78.6%)	293 (56.7%)	147 (73.5%)	161 (77.8%)	311 (85.7%)	966 (87.7%)	

Abbreviations: ER, estrogen receptor; HER2, human epidermal growth factor receptor 2; PR, progesterone receptor.

**FIGURE 1:**
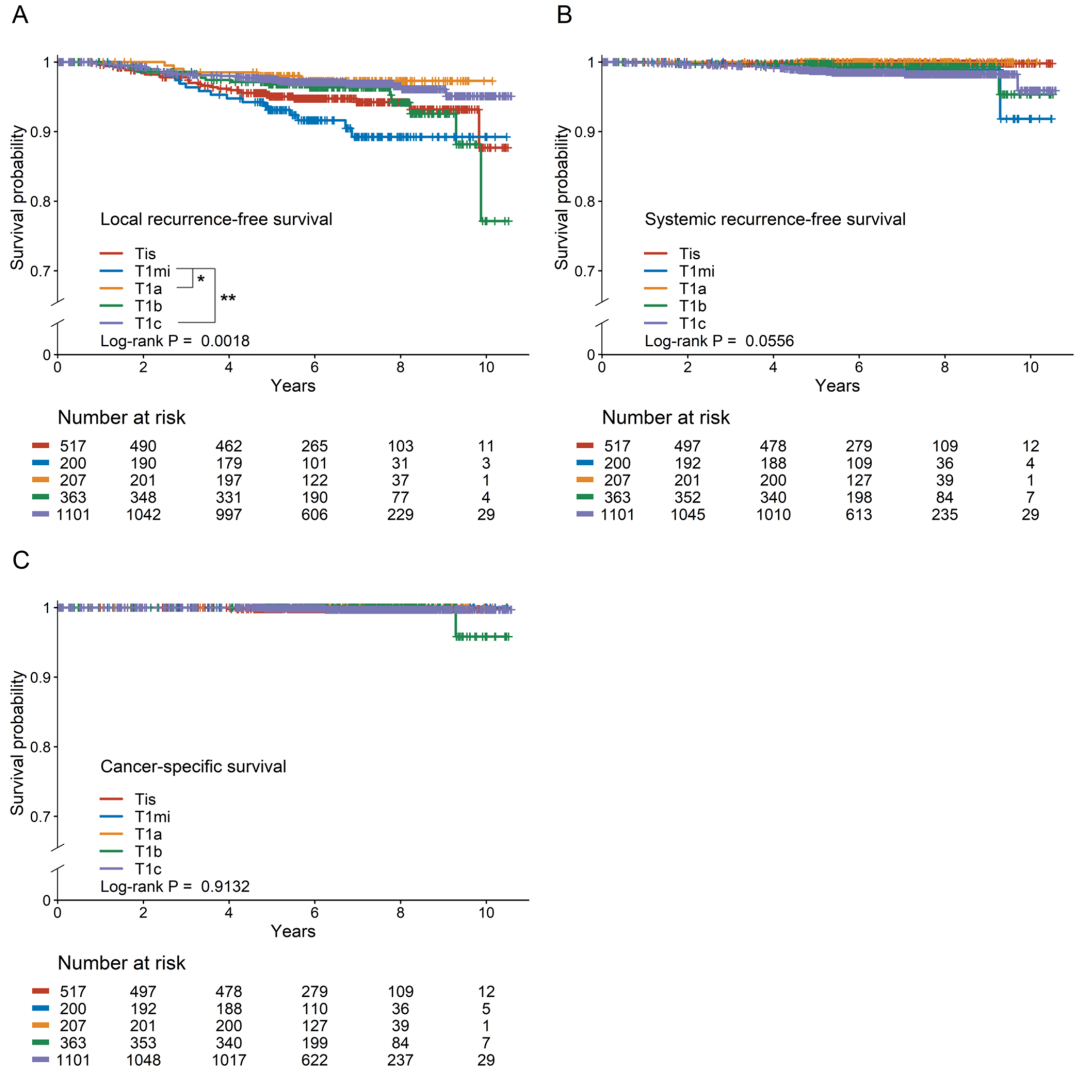
Kaplan–Meier survival curves according to T stage. (A) Local recurrence‐free survival MIBC showed significantly poorer LRFS compared to T1a and T1c tumors. *p < 0.05; **p < 0.001. (B) Systemic recurrence‐free survival. There was no significant difference in SRFS according to T stages. (C) Cancer‐specific survival. There was no significant difference in CSS according to T stages. CSS, cancer‐specific survival; LRFS, local recurrence‐free survival; SRFS, systemic recurrence‐free survival.

Kaplan–Meier curves comparing survival according to T stage are presented in Figure 1. The median follow‐up period was 73 months (range, 1–126 months). During the entire study period, 17 (8.5%) LRs occurred in 200 patients with MIBC, while 28 (5.4%) and 55 (3.3%) cases of LR occurred in patients with CIS and T1a–c tumors, respectively. The 5‐year LRFS rates were 95.0%, 93.1%, 98.0%, 96.8%, and 97.6% for patients with Tis, T1mi, T1a, T1b, and T1c stages. Patients with MIBC showed significantly worse LRFS than those with T1a and T1c tumors (*p* = 0.002) (Figure 1A). However, only 24 systemic recurrences and four cancer‐specific deaths were observed during the study period, and there was no difference in SRFS and CSS according to T stage (SRFS, *p* = 0.056; CSS, *p* = 0.913) (Figure 1B,C).

**FIGURE 2:**
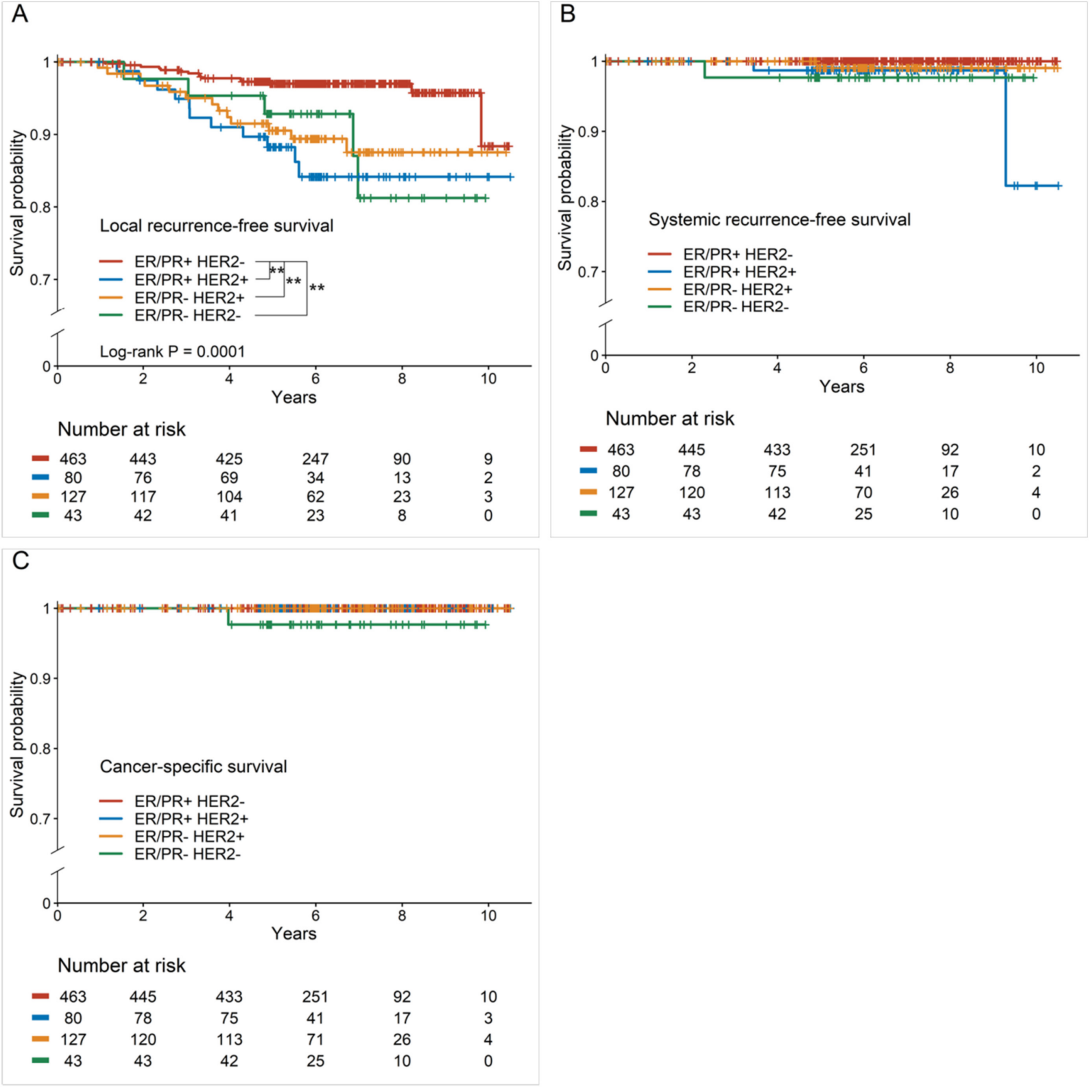
Kaplan–Meier survival curves according to cancer subtype in patients with CIS and MIBC. (A) Local recurrence‐free survival. Patients with ER/PR+, HER2− subtypes showed significantly higher LRFS compared to patients with other cancer subtypes. **p < 0.001. (B) Systemic recurrence‐free survival. Systemic recurrences were observed in 4 patients. The difference in SRFS according to cancer subtypes was not clinically significant. (C) Cancer‐specific survival. Cancer‐specific deaths were observed in 1 patient. The difference in CSS according to cancer subtypes was not clinically significant. CSS, cancer‐specific survival; ER, estrogen receptor; HER2, human epidermal growth factor receptor 2; LRFS, local recurrence‐free survival; PR, progesterone receptor; SRFS, systemic recurrence‐free survival.

Survival outcomes according to the cancer subtype were compared in patients with CIS or MIBC, both of which contained CIS as the main component. In this cohort, patients with ER/PR+ and HER2− subtypes showed significantly higher LRFS than those with other cancer subtypes (*p* = 0.0001) (Figure 2A). Although SRFS and CSS demonstrated significant differences, clinical significance was not established due to limited number of events; four and one occurrences, respectively (Figure 2B,C).

**FIGURE 3:**
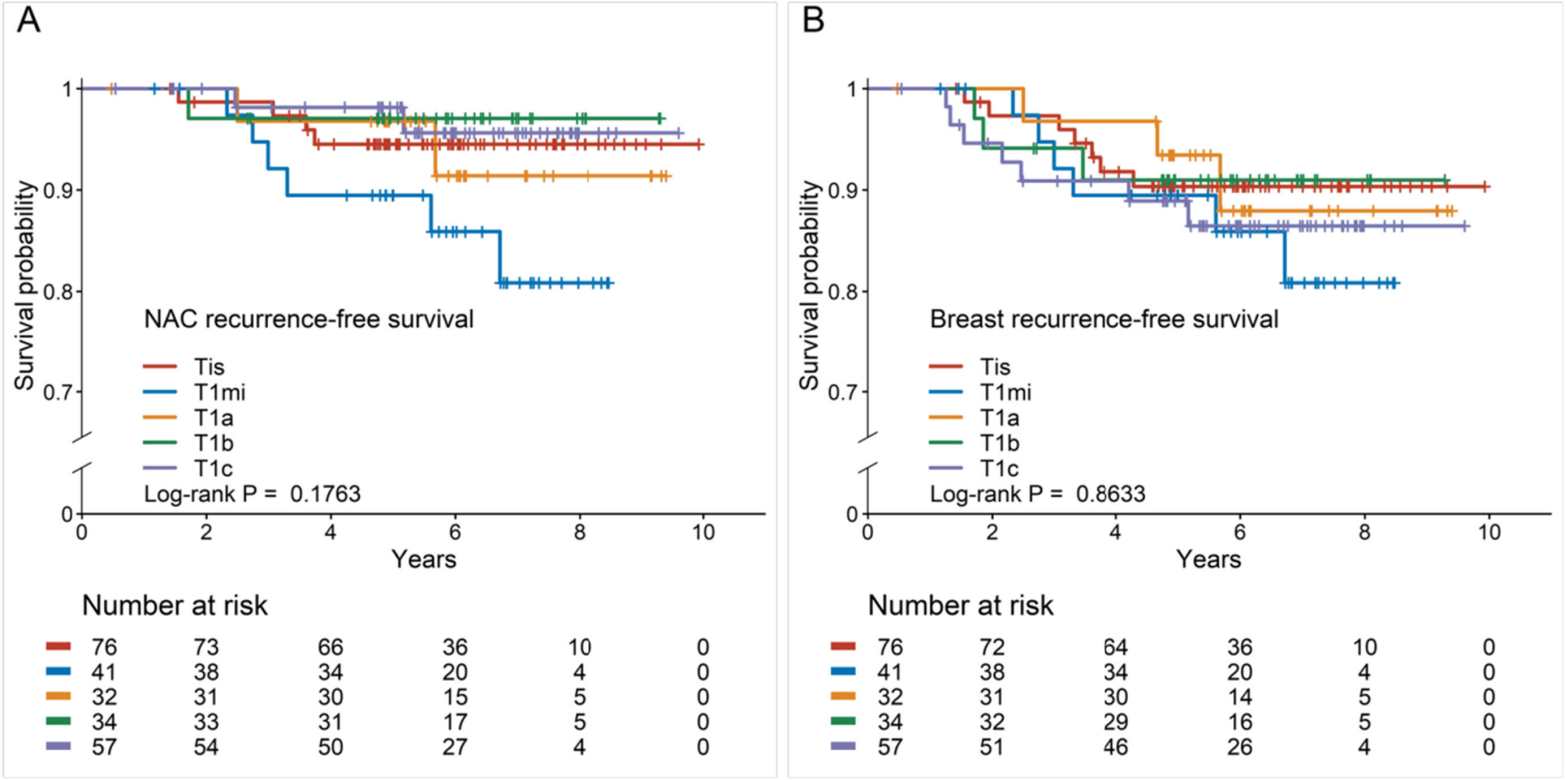
Kaplan–Meier survival curves according to T stage after NAC‐sparing mastectomy. (A) NAC recurrence‐free survival. There was no significant difference in NAC recurrence‐free survival according to T stage. (B) Breast recurrence‐free survival. There was no significant difference in breast recurrence‐free survival according to T stage. NAC, nipple‐areolar complex.

We also compared LRs that occurred on the nipple‐areolar complex (NAC) and the whole breast according to T stage in patients who underwent NAC‐sparing mastectomy (NSM). The recurrence rate in the NAC was higher in patients with MIBC, although not significant (Figure 3A). There was no difference in breast recurrence‐free survival according to T stage (Figure 3B).

A Cox proportional hazard model was constructed to identify the prognostic factors associated with LRFS (Table 3). In univariable analysis, the type of surgery, tumor multiplicity, tumor size, pathological T stage, Ki‐67, cancer subtype, adjuvant endocrine therapy, and adjuvant radiation therapy were significantly associated with LR. In the multivariable analysis using these factors, multiple tumors (hazard ratio (HR) 1.833, 95% confidence interval (CI): 1.117–3.008, *p* = 0.017), high Ki‐67 (HR 1.749, 95% CI: 1.072–2.855, *p* = 0.025), ER/PR+ and HER2+ subtype (HR 1.977, 95% CI: 1.062–3.681, *p* = 0.032), adjuvant endocrine therapy (HR 0.374, 95% CI: 0.198–0.708, *p* = 0.003), and adjuvant radiation therapy (HR 0.225, 95% CI: 0.147–0.343, *p* < 0.0001) were identified as prognostic factors for LR.

**TABLE 3:** Cox proportional hazards model of local recurrence‐free survival for total study population.

Characteristics	Univariable			Multivariable		
	HR	95% CI	*p*	HR	95% CI	*p*
Menopausal status						
Premenopausal	1 (ref)					
Postmenopausal	0.790	0.528–1.181	0.2504			
Type of surgery						
BCS	1 (ref)		**< 0.0001**			
TM	3.410	1.633–7.120	0.0011			
NSM	4.515	2.902–7.026	< 0.0001			
Tumor multiplicity						
Single	1 (ref)			1 (ref)		
Multiple (≥ 2)	1.977	1.211–3.228	**0.0064**	1.833	1.117–3.008	**0.0166**
Tumor size^a^	1.276	1.158–1.406	**< 0.0001**			
Pathological T stage						
T1mi	1 (ref)		**0.0029**			
Tis	0.628	0.344–1.148	0.1306			
T1a	0.276	0.102–0.747	0.0113			
T1b	0.536	0.274–1.05	0.0693			
T1c	0.342	0.19–0.614	0.0003			
Ki‐67 (*n* = 2370)						
≤ 14%	1 (ref)			1 (ref)		
> 14%	2.101	1.368–3.226	**0.0007**	1.749	1.072–2.855	**0.0253**
Subtype (*n* = 2384)						
ER/PR+ HER2−	1 (ref)		**< 0.0001**	1 (ref)		**0.0338**
ER/PR+ HER2+	2.538	1.427–4.513	< 0.0001	1.977	1.062–3.681	0.0316
ER/PR− HER2+	3.088	1.873–5.091	< 0.0001	0.843	0.392–1.816	0.6632
ER/PR− HER2−	1.588	0.827–3.046	0.1644	0.600	0.247–1.455	0.2582
Chemotherapy						
No	1 (ref)					
Yes	0.737	0.455–1.193	0.2138			
Endocrine therapy						
No	1 (ref)			1 (ref)		
Yes	0.329	0.222–0.487	**< 0.0001**	0.374	0.198–0.708	**0.0025**
Anti‐HER2 therapy						
No	1 (ref)					
Yes	0.909	0.422–1.96	0.8083			
Radiation therapy						
No	1 (ref)			1 (ref)		
Yes	0.190	0.128–0.282	**< 0.0001**	0.225	0.147–0.343	**< 0.0001**

*Note:* The *p*‐values that are statistically significant have been highlighted in bold.

Abbreviations: BCS, breast‐conserving surgery; CI, confidence interval; ER, estrogen receptor; HER2, human epidermal growth factor receptor 2; HR, hazard ratio; NSM, nipple‐sparing mastectomy; PR, progesterone receptor; ref, reference; TM, total mastectomy.

^a^ Tumor size was measured as the size of the invasive focus in T1a–c tumors, and as the size of the largest lesion, either invasive focus or in situ component, in Tis and T1mi tumors.

For MIBC patients, despite concerns about overfitting, the type of surgery, tumor multiplicity, tumor size, and adjuvant radiation therapy showed significant association with LR in univariable analysis. In the multivariable analysis, only the type of surgery and tumor multiplicity were identified as significant factors (Table S1).

## 4 Discussion

In this study, we compared the prognostic outcomes of patients with MIBC with CIS or T1a–c invasive breast cancer. There was no difference in SRFS and CSS according to T stage; however, patients with MIBC showed poorer LRFS than those with T1a or T1c tumors. Additionally, Cox regression analysis revealed that tumor multiplicity, Ki‐67 index, cancer subtype, adjuvant endocrine therapy, and adjuvant radiation therapy were significantly associated with LRFS.

MIBC was defined as invasive breast carcinoma with no focus more than 0.1 cm in the great dimension throughout the study, and all patients with MIBC, regardless of the presence or absence of DCIS, were included. Since most MIBC cases are generally associated with DCIS, the characteristics of pure MIBC remain poorly understood. Therefore, we compared the characteristics of patients with pure MIBC to those with MIBC found in DCIS (Table S2). In our data, 15 out of 200 patients (7.5%) had pure MIBC without a DCIS component. Except for Ki‐67 as a continuous variable, no other variables showed significant differences between the two groups. Moreover, classifying pure MIBC as an independent category raises concerns regarding its clinical significance owing to the rarity of the disease. Based on these findings, we did not consider the two groups as distinct entities and analyzed them within the same category.

The 10‐year LRFS rates of the present study were as follows: Tis, 87.7%; T1mi, 89.2%; T1a, 97.3%; T1b, 77.1%; and T1c, 95.1%. These rates differ from the 5‐year LRFS rates because of the limited number of patients with follow‐up periods exceeding 10 years. Based on the 5‐year LRFS or overall trends, the survival rates were in the order of T1a > T1c > T1b > Tis > T1mi. Patients with CIS as the main component, such as Tis or T1mi tumors, showed a higher LR than those with early invasive cancers. Although tumor size generally has a positive relationship with LR, showing a poorer prognosis in breast cancer [11, 15], this study observed an inverse relationship between LRFS and T1b and T1c tumors. This result was attributed to the higher administration of adjuvant chemotherapy and anti‐HER2 therapy in patients with T1c tumors (Table 2). However, compared to patients with CIS or T1a tumors who did not receive chemotherapy or targeted therapy, patients with MIBC still showed a trend of higher LR, suggesting the characteristics of the MIBC entity.

While studies on the prognosis of patients with MIBC are limited and the results are inconsistent, several previous studies have shown a higher LR in patients with MIBC than in those with DCIS or T1‐2 tumors [12, 13, 16]. Goldberg et al. [12] compared the long‐term outcomes of patients with MIBC with T1a‐T2 tumors and revealed that the 10‐year LR rate, including both invasive and noninvasive LR, was higher in those with MIBC at 22.6% compared to those with T1a‐T2 tumors (6.9%). Shiino et al. [13] conducted a meta‐analysis regarding the prognosis of MIBC and reported significantly shorter disease‐free survival (DFS) for patients with MIBC compared to those with pure DCIS (HR, 1.58; 95% CI: 1.10–2.28; *p* = 0.01).

Cancer subtype can be one of the factors contributing to the LR rate of MIBC. In general, for breast cancer, hormone receptor (HR)‐positive and HER2‐negative tumors are most frequent, comprising 68%–87% of tumors, while HR‐positive and HER2‐positive tumors, and HR‐negative and HER2‐positive tumors account for approximately 10%–12% and 4%–6% of tumors, respectively [3, 17]. However, for MIBC in the present study, patients with HR‐positive and HER2‐negative tumors showed a lower proportion (40.0%), and those with HER2‐positive tumors, either HR‐positive or HR‐negative, showed a much higher proportion than patients with breast cancer with other T stages. Previous studies have reported similar receptor expression in MIBC [18, 19, 20]. As already well known, survival, both CSS and DFS, differs according to cancer subtype in invasive breast cancer [21, 22]. Therefore, we compared survival according to the cancer subtype among patients with CIS as the main component and found a significant difference in LRFS (*p* = 0.0001) (Figure 2). Our results were consistent with previous studies which identified the molecular subtype as a prognostic factor for CSS or DFS [7, 11, 13, 20]. In some studies, the findings suggest that the benefit of adjuvant chemotherapy varies according to the receptor status in patients with MIBC [23, 24]. However, further prospective studies are necessary to evaluate treatment outcomes and prognosis according to cancer subtype.

Owing to growing interest in cosmesis and oncoplastic surgery, NSM has gained wide acceptance as an alternative to standard total mastectomy for breast cancer treatment [25]. The oncologic safety of NSM has been well established [26, 27]; however, we additionally compared recurrence‐free survivals according to T stage in patients who underwent NSM in our study cohort to assess the impact of NSM on the LR rate of MIBC. We found no difference in NAC and breast recurrence‐free survival according to T stage after NSM; therefore, NSM can be considered a safe treatment option for patients with MIBC, similar to patients with other T stages. Few studies have explored the effect of the type of surgery on prognostic outcomes. Several studies have compared the clinical outcomes between BCS and mastectomy in patients with MIBC and reported no difference in prognostic outcomes according to the type of surgery, especially with proper adjuvant treatment [23, 28]. However, owing to the lack of research on the safety of NSM compared with total mastectomy in patients with MIBC, additional studies with long‐term outcomes are required for appropriate treatment guidance.

We also constructed a Cox regression hazard model and identified multiple tumors, high Ki‐67, ER/PR+ and HER2+ subtype, adjuvant endocrine therapy, and adjuvant radiation therapy as independent prognostic factors. Pathological T stage was a significant factor in univariable analysis; however, it was not significant in the multivariable analysis. Based on this result, factors other than T stage may have a greater impact on LR in patients with early stage breast cancer. Additionally, we performed Cox regression analysis specifically for patients with MIBC, identifying the type of surgery and tumor multiplicity as prognostic factors (Table S1). However, the generalization of this finding to other cohorts is limited because of the considerable risk of overfitting. In previous studies involving patients with MIBC, microinvasion, tumor size, tumor grade, and receptor status were identified as prognostic factors for LR [7, 10, 11, 20]. Receptor status was consistently found to be a common factor in both our study and previous research [7, 11, 20], suggesting a significant role for receptor status in LR in patients with MIBC.

This is one of the large‐scale studies on the prognostic outcomes of MIBC conducted in Korea, and has the strength of concurrently comparing the survival of patients with MIBC with that of patients with CIS and T1 invasive breast cancer. The results of this study can provide information on the characteristics and clinical outcomes of MIBC, as well as insights into which disease, either CIS or invasive cancer, shares a more similar entity in terms of prognosis. However, this study had some limitations. First, there was selection bias due to the retrospective nature of the study. The distribution of patients at each T stage varied, potentially influencing the outcomes, particularly in patients with MIBC with a relatively small sample size. Additionally, the administered adjuvant treatments were not consistent across T stages because patients in this study were treated based on current guidelines and clinician discretion; thus, we could not exclude the impact of treatments on survival. Second, the median follow‐up period was relatively short when comparing long‐term outcomes. Despite the study having a median follow‐up period of over 5 years, the incidence of events during this period was low, as the study only included patients with early stage breast cancer. A follow‐up study using long‐term data (> 10 years) is required for more accurate analysis. Finally, the clinicopathological data used for the Cox regression analysis in this study were limited. Since the study was designed for survival analysis, other pathological factors, such as margin status, number of microinvasive foci, lymphovascular invasion, CK5/6 expression, and comedo necrosis, which have been previously identified as prognostic factors for MIBC [13, 19, 29, 30], were not assessed. Further studies analyzing the association between these factors and LR in patients with MIBC are warranted.

## 5 Conclusions

In conclusion, the risk of LR was higher in patients with MIBC than in those with small invasive breast cancer, although there was no significant difference in the risk of systemic recurrence. Therefore, if indicated, adjuvant endocrine and radiation therapies should be administered to prevent undertreatment, and proactive follow‐up should be conducted to detect LR in patients with MIBC.

## Author Contributions

**Ran Song:** conceptualization (equal), investigation (equal), methodology (equal), writing – original draft (lead). **Dong‐Eun Lee:** formal analysis (lead), writing – review and editing (equal). **So‐Youn Jung:** writing – review and editing (equal). **Seeyoun Lee:** writing – review and editing (equal). **Han‐Sung Kang:** writing – review and editing (equal). **Jai Hong Han:** writing – review and editing (equal). **Jaeyeon Woo:** writing – review and editing (equal). **Eun‐Gyeong Lee:** conceptualization (equal), funding acquisition (lead), investigation (equal), methodology (equal), supervision (lead), writing – review and editing (equal).

## Ethics Statement

This study was approved by the Institutional Review Board (IRB) of the National Cancer Center, Korea (IRB No. NCC2022‐0275).

## Consent

The need for informed consent was waived due to the retrospective nature of the study.

## Conflicts of Interest

The authors declare no conflicts of interest.

## Supporting information


**Table S1.** Cox proportional hazards model of local recurrence‐free survival for patients with MIBC.
**Table S2.** Comparison of baseline characteristics between patients with pure MIBC and MIBC with DCIS.

## Data Availability

The data presented in this study are available from the corresponding author upon reasonable request. The data are not publicly available due to privacy policies.
