# Supplementary material for: Unveiling the Clinical Path of Microinvasive Breast Cancer: A Comparative Study With Tis‐T1 Breast Cancer
Source: Cancer Med. 2024 Oct 9;13(19):e70297. doi: 10.1002/cam4.70297 (PMC11462587; doi:10.1002/cam4.70297)
Supplement: Supplementary file 1 — Table S1. Cox proportional hazards model of local recurrence‐free survival for patients with MIBC. Table S2. Comparison of baseline characteristics between patients with pure MIBC and MIBC with DCIS. [file CAM4-13-e70297-s001.docx]

**Supplementary Table 1. Cox proportional hazards model of local recurrence-free survival for patients with MIBC**

| **Characteristics** | **Univariable** | | | **Multivariable** | | |
| --- | --- | --- | --- | --- | --- | --- |
|  | **HR** | **95% CI** | ***p*** | **HR** | **95% CI** | ***p*** |
| **Age** |  |  |  |  |  |  |
| ≤40 years | 1 (ref) |  |  |  |  |  |
| >40 years | 0.738 | 0.241–2.265 | 0.5960 |  |  |  |
| **Menopausal status** |  |  |  |  |  |  |
| Premenopausal | 1 (ref) |  |  |  |  |  |
| Postmenopausal | 0.421 | 0.148–1.197 | 0.1046 |  |  |  |
| **Type of surgery** |  |  |  |  |  |  |
| BCS | 1 (ref) |  | **0.0096** | 1 (ref) |  | **0.010** |
| TM | 6.987 | 1.822–26.803 | 0.0046 | 6.529 | 1.702–25.046 | 0.0062 |
| NSM | 2.981 | 1.034–8.599 | 0.0433 | 3.22 | 1.113–9.316 | 0.0310 |
| **Tumor multiplicity** |  |  |  |  |  |  |
| Single | 1 (ref) |  |  | 1 (ref) |  |  |
| Multiple (≥2) | 4.170 | 1.468–11.844 | **0.0073** | 4.282 | 1.496–12.259 | **0.0067** |
| **Tumor size (cm)*** | 1.320 | 1.073-1.622 | **0.0095** |  |  |  |
| **Histologic grade (n=187)** |  |  |  |  |  |  |
| 1–2 | 1 (ref) |  |  |  |  |  |
| 3 | 2.339 | 0.85–6.438 | 0.100 |  |  |  |
| **Ki-67** |  |  |  |  |  |  |
| ≤14% | 1 (ref) |  |  |  |  |  |
| >14% | 3.466 | 0.793–15.154 | 0.0986 |  |  |  |
| **Subtype** |  |  |  |  |  |  |
| ER/PR+ HER2- | 1 (ref) |  | 0.0895 |  |  |  |
| ER/PR+ HER2+ | 7.899 | 1.594–39.149 | 0.0114 |  |  |  |
| ER/PR- HER2+ | 3.999 | 0.83–19.273 | 0.0841 |  |  |  |
| ER/PR- HER2- | 4.41 | 0.62–31.347 | 0.1381 |  |  |  |
| **Endocrine therapy** |  |  |  |  |  |  |
| No | 1 (ref) |  |  |  |  |  |
| Yes | 0.565 | 0.215–1.487 | 0.2475 |  |  |  |
| **Radiation therapy** |  |  |  |  |  |  |
| No | 1 (ref) |  |  |  |  |  |
| Yes | 0.334 | 0.129–0.866 | **0.0241** |  |  |  |

* Tumor size was measured as the size of the largest lesion, either invasive focus or in situ component.
MIBC, microinvasive breast cancer; HR, hazard ratio; CI, confidence interval; ref, reference; BCS, breast-conserving surgery; TM, total mastectomy; NSM, nipple-sparing mastectomy; ER, estrogen receptor; PR, progesterone receptor; HER2, human epidermal growth factor receptor 2

**Supplementary Table 2. Comparison of baseline characteristics between patients with pure MIBC and MIBC with DCIS**

| **Characteristics** | **Total MIBC (n=200)** | **Pure MIBC (n=15)** | **MIBC with DCIS (n=185)** | ***p*** |
| --- | --- | --- | --- | --- |
| **Age** |  |  |  |  |
| Median (min-max) | 50 (27-77) | 48 (32-63) | 50 (27-77) | 0.2876 |
| ≤40 years | 36 (18%) | 3 (20%) | 33 (17.8%) | 0.7365 |
| >40 years | 164 (82%) | 12 (80%) | 152 (82.2%) |  |
| **Menopausal status** |  |  |  | 0.7575 |
| Premenopausal | 99 (49.5%) | 8 (53.3%) | 91 (49.2%) |  |
| Postmenopausal | 101 (50.5%) | 7 (46.7%) | 94 (50.8%) |  |
| **Type of surgery** |  |  |  | 0.3635 |
| BCS | 146 (73%) | 13 (86.7%) | 133 (71.9%) |  |
| Mastectomy | 54 (27%) | 2 (13.3%) | 52 (28.1%) |  |
| **Tumor multiplicity** |  |  |  | 0.3714 |
| Single | 180 (90%) | 15 (100%) | 165 (89.2%) |  |
| Multiple (≥2) | 20 (10%) | 0 | 20 (10.8%) |  |
| **Histologic grade (n=187)** |  |  |  | 0.1530 |
| 1 | 14 (7%) | 1 (6.7%) | 13 (7%) |  |
| 2 | 91 (45.5%) | 8 (53.3%) | 83 (44.9%) |  |
| 3 | 82 (41%) | 2 (13.3%) | 80 (43.2%) |  |
| **Ki-67** |  |  |  |  |
| Median (min-max) | 23.0 (1.0-67.0) | 12.0 (1.0-34.0) | 24.0 (1.0-67.0) | 0.0144 |
| ≤14% | 60 (30%) | 8 (53.3%) | 52 (28.1%) | 0.0739 |
| >14% | 140 (70%) | 7 (46.7%) | 133 (71.9%) |  |
| **Subtype** |  |  |  | 0.5898 |
| ER/PR+ HER2- | 80 (40.0%) | 8 (53.3%) | 72 (38.9%) |  |
| ER/PR+ HER2+ | 34 (17.0%) | 3 (20%) | 31 (16.8%) |  |
| ER/PR- HER2+ | 70 (35.0%) | 4 (26.7%) | 66 (35.7%) |  |
| ER/PR- HER2- | 16 (8.0%) | 0 | 16 (8.7%) |  |
| **Chemotherapy** |  |  |  | - |
| No | 200 (100%) | 15 (100%) | 185 (100%) |  |
| Yes | 0 | 0 | 0 |  |
| **Endocrine therapy** |  |  |  | 0.1860 |
| No | 88 (44.0%) | 4 (26.7%) | 84 (45.4%) |  |
| Yes | 112 (56.0%) | 11 (73.3%) | 101 (54.6%) |  |
| **Anti-HER2 therapy** |  |  |  | - |
| No | 200 (100%) | 15 (100%) | 185 (100%) |  |
| Yes | 0 | 0 | 0 |  |
| **Radiation therapy** |  |  |  | 0.1233 |
| No | 53 (26.5%) | 1 (6.7%) | 52 (28.1%) |  |
| Yes | 147 (73.5%) | 14 (93.3%) | 133 (71.9%) |  |

MIBC, microinvasive breast cancer; DCIS, ductal carcinoma in situ; min, minimum; max, maximum; BCS, breast-conserving surgery; ER, estrogen receptor; PR, progesterone receptor; HER2, human epidermal growth factor receptor 2
